# Differential Distribution of Brain Metastases from Non-Small Cell Lung Cancer Based on Mutation Status

**DOI:** 10.3390/brainsci13071057

**Published:** 2023-07-11

**Authors:** Bihong T. Chen, Taihao Jin, Ningrong Ye, Sean W. Chen, Russell C. Rockne, Stephanie Yoon, Isa Mambetsariev, Ebenezer Daniel, Ravi Salgia

**Affiliations:** 1 Department of Diagnostic Radiology, City of Hope National Medical Center, 1500 East Duarte Road, Duarte, CA 91010, USAedaniel@coh.org (E.D.); 2 Department of Medical Oncology and Therapeutics Research, City of Hope Comprehensive Cancer Center and Beckman Research Institute, Duarte, CA 91010, USAimambetsariev@coh.org (I.M.); rsalgia@coh.org (R.S.); 3 Division of Mathematical Oncology, City of Hope National Medical Center, 1500 East Duarte Road, Duarte, CA 91010, USA; rrockne@coh.org; 4 Department of Radiation Oncology, City of Hope National Medical Center, 1500 East Duarte Road, Duarte, CA 91010, USA; styoon@coh.org

**Keywords:** brain metastases, lung cancer, mutation status, spatial distribution

## Abstract

Non-small cell lung cancer (NSCLC) has a high rate of brain metastasis. The purpose of this study was to assess the differential distribution of brain metastases from primary NSCLC based on mutation status. Brain MRI scans of patients with brain metastases from primary NSCLC were retrospectively analyzed. Brain metastatic tumors were grouped according to mutation status of their primary NSCLC and the neuroimaging features of these brain metastases were analyzed. A total of 110 patients with 1386 brain metastases from primary NSCLC were included in this study. Gray matter density at the tumor center peaked at ~0.6 for all mutations. The median depths of tumors were 7.9 mm, 8.7 mm and 9.1 mm for EGFR, ALK and KRAS mutation groups, respectively (*p* = 0.044). Brain metastases for the EGFR mutation-positive group were more frequently located in the left cerebellum, left cuneus, left precuneus and right precentral gyrus. In the ALK mutation-positive group, brain metastases were more frequently located in the right middle occipital gyrus, right posterior cingulate, right precuneus, right precentral gyrus and right parietal lobe. In the KRAS mutation-positive patient group, brain metastases were more frequently located in the posterior left cerebellum. Our study showed differential spatial distribution of brain metastases in patients with NSCLC according to their mutation status. Information regarding distribution of brain metastases is clinically relevant as it could be helpful to guide treatment planning for targeted therapy, and for predicting prognosis.

## 1. Introduction

Non-small cell lung cancer (NSCLC) has high rate of brain metastasis (BM), with 10–20% diagnosed with BM at the initial stage and 40% diagnosed during their course of disease [1]. The prognosis is generally poor in NSCLC patients with BM [2,3]. Surgical resection, stereotactic radiosurgery (SRS) and whole-brain radiotherapy (WBRT) have been the conventional treatment methods for BM [4]. Improved systemic therapies with improved penetration into the central nervous system have considerably increased the overall survival and quality of life for patients with BM [5]. The knowledge of spatial distribution of BM is critical for selecting treatment strategies and predicting prognosis. For example, treatment for low volume of BM can focus on the tumor to avoid radiation to brain regions less likely for BM [6]. This strategy would be helpful to adequately treat the BM but also to preserve brain tissue for maintaining function and quality of life in patients with cancer. 

Distribution of BM is non-uniform and may be different according to the mutation status. Epidermal growth factor receptor (EGFR), anaplastic lymphoma kinase (ALK) and v-Ki-ras2 Kirsten rat sarcoma viral oncogene (KRAS) are the most common mutated oncogenes associated with lung cancer BM [7]. Takano et al. assessed the spatial distribution of BM on brain MRI and CT images of 200 NSCLC patients with 1033 brain metastatic tumors. They reported that BM from NSCLC with EGFR L858R mutation occurred more frequently in the cerebellum, caudate nucleus and temporal lobe than those with EGFR exon 19 deletion. However, their study did not assess spatial distribution of BM in patients with ALK or KRAS mutation [8].

BM is known to occur in watershed areas and gray–white matter junction because of the distribution of small vessels [6,9]. A study by Yanagihara et al. studied the spatial distribution of 150 BM tumors in 28 patients with lung or breast cancer. They reported that 70–80% of lesions were located in the cerebral hemisphere, 15% to 20% in the cerebellum, and 3% in the brainstem [6]. However, their sample size was small and they did not consider mutation status in their study [6]. Characteristics of BM such as tumor size, volume, depth from the brain surface, and peritumoral edema are important in assessing treatment response and prognosis [10,11]. Prior studies have reported that increases in tumor size and depth indicate poor prognosis in patients with BM [12,13]. Tumor size has an important role in surgical management and survival prediction in patients with BM [14]. A study by Bennett et al. assessed the prognosis of 584 patients with a single BM receiving stereotactic radiosurgery treatment. They reported that tumor volume was associated with overall survival in patients with BM [15]. Calluaud et al. evaluated 120 patients who underwent surgical resection for BM in posterior fossa and reported that high peritumoral edema/tumor ratio was associated with poor prognosis [16]. However, few studies have comprehensively assessed spatial distribution and tumor characteristics of BM according to mutation status.

In this study, we analyzed brain MRI images of patients with BM from primary NSCLC and assessed the distribution of BM according to their mutational status. In addition, we also assessed the distribution of gray matter density (GMD) and additional neuroimaging characteristics including the number, size, depth of BM and peritumoral edema/tumor volume ratio according to mutation status. We hypothesized that BM tumors from NSCLC were preferentially distributed in the specific regions of the brain according to the mutation status of primary lung cancer. To test this hypothesis, we performed location analysis and generated distribution frequency maps for EGFR, ALK or KRAS mutation-positive groups. We then evaluated BM frequencies to identify the unique spatial distribution pattern for each mutation group by comparing BM frequencies to uniform random distribution for each mutation group. Information on differential distribution of BM for specific mutations should be helpful for treatment planning and allow for more precise irradiation for lung cancer BM.

## 2. Material and Methods

### 2.1. Clinical Data

We retrospectively identified a cohort of patients with NSCLC and brain metastases from 2009 to 2017 in the lung cancer database at City of Hope National Medical Center (Duarte, CA). The inclusion criteria for the patients were as follows: having NSCLC as the primary cancer type, having at least one of the three mutations, i.e., either EGFR, ALK or KRAS, having pre-treatment brain MRI scans containing the three-dimensional (3D) T1-weighted contrast-enhanced images (T1c) and Fluid Attenuated Inversion Recovery (FLAIR) images. Patients were excluded from this study if their brain MRI scans were suboptimal for assessment. The suboptimal brain MRI scans were defined as the scans with significant motion artifacts which rendered postprocessing inadequate, or susceptibility artifacts such as the artifacts from metal or calcifications which obscured signal intensity of the tumors, or the scans without 3D post-contrast images for tumor contouring and tumor registration on the Montreal Neurological Institute (MNI) standard space. The patients were grouped according to their mutation status: EGFR, ALK, or KRAS (Table 1). The details of patients’ information have been reported in our previous studies focusing on radiomic prediction of mutation status and survival in patients with BM from NSCLC [17,18]. Clinical and demographical information including gender, age, smoking, race and metastatic sites was collected for the cohort (Table 1). The patients’ clinical data were abstracted from the electronic medical record. This study was approved by the Institutional Review Board at our institution (IRB#17369). Written informed consent was waived for this retrospective study.

### 2.2. Tumor Segmentation and Registration

**Figure 1:**
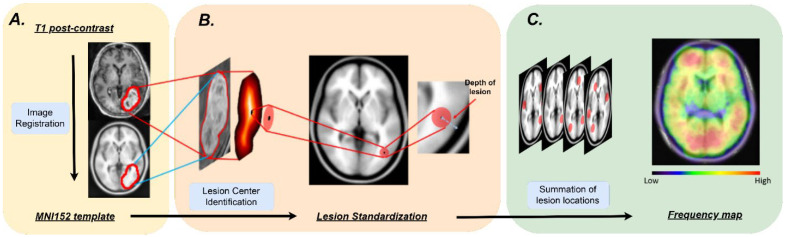
A schema presenting the process of tumor frequency map construction. (**A**) Registration of an original T1-weighted post contrast (T1c) image to the standard space in the Montreal Neurological Institute (MNI) template (the MNI 152). (**B**) Lesion center identification, lesion depth measurement and lesion standardization. The center of the lesion was identified by locating the voxel with maximal distance from the lesion surface using morphological analysis (see Methods), and the lesion was then visualized by creating a 20-mm diameter sphere around the lesion center. (**C**) Construction of tumor frequency map by summation of all 20 mm diameter lesion spheres for each patient.

Brain tumor segmentation was manually performed on the T1c and FLAIR images under the supervision of the study neuroradiologist (BTC) using ITK snap (http://www.itksnap.org/pmwiki/pmwiki.php, accessed on 5 May 2019). Image registration was performed using the diffeomorphic anatomical registration through exponentiated lie algebra (DARTEL) toolbox in the Statistical Parametric Mapping software version 12 (SPM 12) (Wellcome Trust Centre for Neuroimaging, London, UK). Specifically, the following processing was performed. First, we segmented each T1c image of the BM to generate the tissue probability maps for gray matter, white matter and cerebrospinal fluid. Second, we utilized the DARTEL tool [19] to improve the inter-subject alignment. Third, we normalized the gray matter probability maps to the standard space, i.e., the Montreal Neurological Institute (MNI) template tool [19]. Finally, we transformed the segmented tumor masks into the MNI template using the transformation parameters generated by the second and third steps. All T1c images were manually adjusted to reset the origin at the anterior commissure and to set the posterior commissure at the second axis of the coordinate system. Figure 1 presents the process of tumor frequency map construction and Figure 1A shows a representative image registration from the original T1c image to the standard space in the MNI152 template. As indicated in Figure 1A, our technique for image registration of tumor masks to the MNI space was satisfactory, with the contoured tumor on the T1c image being accurately registered onto the MNI152 template. The registration results of all tumor masks were also visually inspected via similar approach by the three trained imaging researchers (TJ, NY and BTC).

### 2.3. Construction of Tumor Frequency Maps

The brain parenchymal mask was constructed with the following steps. First, we applied a threshold of 0.1 to the brain image obtained by voxel-wise image addition of the gray matter and white matter probability maps included in SPM12. This step created holes (voxel value equals 0) inside the brain parenchyma profile where the combined probability density of gray matter and white matter was less than 0.1, such as the ventricles. Second, these holes were filled by morphological close operation using the image processing toolbox in Matlab (Mathworks, Boston, MA, USA).

We identified the center of a tumor mask as the voxel furthest from the tumor surface by the following two steps: 1. Computing the distance transformation matrix for the complementary binary image of the tumor mask, which was the distance of each voxel in the tumor mask from the tumor surface. 2. Locating the tumor center as the voxel with the largest distance transformation matrix value in the tumor mask. The depth of tumor locations was computed by constructing the distance transformation matrix of the complementary binary image of the brain parenchymal mask. Because the value of this distance transformation matrix at any location was its distance from the parenchymal surface, the depth of a tumor was simply the value of the distance transformation matrix at the tumor center location. The gray matter density (GMD) at the tumor center location was computed by checking the GMD map in the MNI standard space that was created in the process of image registration using DARTEL, as described in the previous section.

The tumor distribution frequency maps for each of the three mutation groups (EGFR, ALK, and KRAS) were constructed using the method described by Takano et al. [8]. First, we constructed a binary image of a 20-mm radial sphere centered at the location of the tumor center for each tumor for each mutation group. Then, we constructed the tumor frequency image through voxel-wise image addition of all binary images, which was normalized by the total number of the tumors for each mutation group. Figure 1 presents the schema of image analysis. A tumor frequency difference map between two mutation groups was constructed through voxel-wise subtraction of the tumor frequency maps of the two mutation groups. We also constructed uniformly random distributed tumor frequency maps to simulate BM distribution for each of the mutation groups by first relocating the tumor center of each patient in the mutation group to a randomly chosen position within the brain parenchyma mask using a uniform random number generator, and then the tumor frequency map was constructed as described above.

### 2.4. Statistical Analysis

The statistical analysis for identification of the brain region where the frequency of BM was higher than that of uniform random distribution was performed through the following steps. First, we determined the voxel-wise threshold such that the probability of identifying suprathreshold voxels in uniformly distributed random tumor maps was equal to or smaller than *p* = 0.001. Second, we determined the cluster-size threshold such that the probability of finding those clusters of suprathreshold voxels larger than the cluster-size threshold in uniformly distributed tumor frequency maps was equal to or smaller than *p* = 0.05. Finally, we applied the voxel-wise threshold to the tumor frequency map of each mutation group and identified those clusters which were larger than the cluster-size threshold as the brain regions where the tumor frequency was statistically significantly higher than that of uniform tumor distribution. To determine the voxel-wise threshold, we generated 10,000 uniformly distributed random tumor frequency maps and the voxel-wise threshold at each voxel was determined as the 1−p quantile value at each voxel using the Matlab (Mathworks, Boston, MA, USA) function quantile (x, 1−p). To determine the cluster-wise threshold, we generated a total of N = 1,000,000 uniformly distributed random tumor frequency maps, and generated clusters by applying the voxel-wise threshold. We set the cluster-wise threshold in such a way that there was a total of p*N clusters (5 in every 100 images) that were larger in size than the cluster-wise threshold. The p values of these clusters were determined by ranking the cluster size among 1,000,000 simulated frequency maps. The computed voxel and cluster level thresholds are listed in the Supplementary Table S1.

Statistical analysis of the demographic and tumor information was performed with the analysis of variance (ANOVA) test using a statistical package in the open-source Scientific Tools for Python library (SciPy, https://www.scipy.org/, accessed on 5 May 2019). We analyzed the mean, standard deviation (SD) and statistical significance of demographic data such as age, gender, race, history of smoking and tumor histology using ANOVA. We also tested the significance of tumor features such as the number of tumors, tumor sizes, edema/tumor ratio and tumor depth using ANOVA.

Three-group Kruskal–Wallis tests were performed on the size of the tumor, depth of the tumor from brain surface and peritumoral edema/tumor ratio among the EGFR, KRAS and ALK mutation-positive groups. Additionally, we performed pairwise comparisons using the Kruskal–Wallis test to detect the statistically significant pairwise difference between mutation groups such as EGFR versus ALK, EGFR versus KRAS, and ALK versus KRAS. In pairwise comparison, we analyzed the significance of tumor features such as the number of tumors, tumor size, peritumoral edema/tumor ratio and tumor depth between the mutation groups. The *p*-values were two-sided, and *p* < 0.05 was considered statistically significant.

## 3. Results

### 3.1. Patient Characteristics

A total of 110 patients with BM from NSCLC were included in our study. Most patients had multiple BM tumors, and a total of 1386 tumors were included in the final analysis, with 1141 tumors being EGFR mutation-positive, 149 being ALK mutation-positive, and 96 being KRAS mutation-positive. Patient information and tumor characteristics are summarized in Table 1. There were statistically significant differences in race (*p* = 0.016) and history of smoking (*p* < 0.001). There was a significantly higher percentage of Asian patients in the EGFR mutation-positive group than the EGFR mutation-negative group (*p* = 0.042). The KRAS mutation-positive group had a statistically higher percentage of smokers than the KRAS mutation-negative group (*p* = 0.0001).

### 3.2. Tumor Characteristics

The number of tumors, tumor size and peritumoral edema/tumor ratio in the three mutation-positive groups of patients are presented in Table 1. There were no significant differences among the three mutation-positive groups in the number of tumors per patient (*p* = 0.34). However, there were significant differences in the mean tumor volumes among the three mutation-positive groups (*p* < 0.0001). Pairwise comparisons indicated that the mean tumor size for the KRAS mutation-positive group was significantly larger than that of the ALK mutation-positive group (*p* = 0.01), which was significantly larger than that of the EGFR mutation-positive group (*p* = 0.001). There were also significant differences in the peritumoral edema/tumor ratios among the three mutation-positive groups (*p* < 0.0001). Pairwise comparison indicted that the edema/volume ratio for the KRAS mutation-positive group was significantly larger than that of the EGFR mutation-positive group (*p* = 0.0003). However, there was no significant difference in the edema/tumor ratio between the ALK- and KRAS-positive groups (*p* = 0.81).

Measurement of tumor depth within the three mutation-positive groups is presented in the Supplementary Figure S1. The median depths were 7.9 mm, 8.7 mm and 9.1 mm for the EGFR, ALK and KRAS mutation-positive groups, respectively. A three-group Kruskal–Wallis test indicated that the difference between the median depths was statistically significant (*p* = 0.044). However, pairwise comparisons using the Kruskal–Wallis test did not detect a statistically significant difference in the measurements of median depth between any pair of the mutation-positive groups. The *p* values for the median depth of EGFR versus ALK, EGFR versus KRAS, and ALK versus KRAS were 0.058, 0.78 and 0.072, respectively.

### 3.3. Distribution of GMD

The peak of GMD distribution for all three mutation-positive groups was located at ~0.6 at a scale of 0 to 1. This GMD distribution indicated that a higher probability of BM occurred at close to the halfway between the brain surface and the midline of the brain, i.e., at the gray–white matter junction, which could also be visually appreciated from the tumor frequency maps presented in Supplementary Figure S2.

### 3.4. Brain Regions with Higher Frequency of BM Than Random Distribution

**Figure 2:**
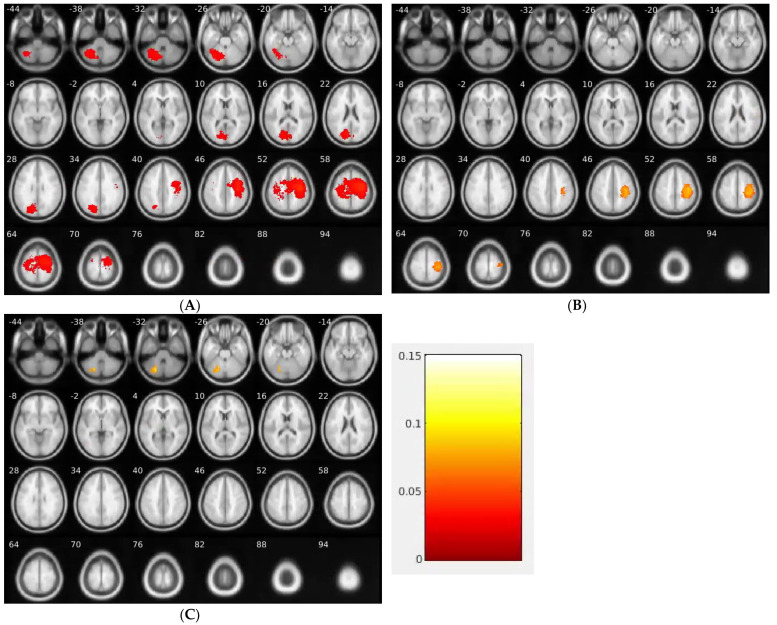
Brain regions where the brain metastases occurred at higher frequency than uniform random distribution for EGFR (**A**), ALK (**B**) and KRAS (**C**) mutation groups. EGFR, epidermal growth factor receptor; ALK, anaplastic lymphoma kinase; KRAS, v-Ki-ras2 Kirsten rat sarcoma viral oncogene.

In the EGFR mutation-positive group, higher tumor-frequency regions consisted of three clusters located in the left cerebellum, part of cuneus in the left occipital lobe and precuneus in the left parietal lobe and the precentral gyrus of the right frontal lobe (Figure 2, Table 2). In the ALK mutation-positive group, there were two clusters with higher tumor frequency. The first cluster included the middle occipital gyrus of the right occipital lobe, the posterior cingulate of the right limbic lobe and the precuneus (Brodmann area (BA) 18) of the right parietal lobe. The second cluster included the precentral gyrus (BA 4) of the right frontal lobe, the inferior parietal lobule (BA 40) and the postcentral gyrus of the right parietal lobe. In the KRAS mutation-positive patient group, there was a single cluster located in the posterior lobe of the left cerebellum.

## 4. Discussion

Our study showed that lung cancer BM was differentially located in the brain according to mutation status. Specifically, BM from both EGFR and ALK groups had a higher frequency of occurring in the right precentral gyrus while BM from both EGFR and KRAS groups had a higher frequency of occurring in the left cerebellum against random distribution. To the best of our knowledge, this is the first study to focus on the differential spatial distribution of BM among the three common mutations in patients with NSCLC. Our results should motivate further investigation of the mechanism for primary lung cancer metastasizing to the brain.

Our finding of preferential distribution of BM in the cerebellum in NSCLC patients with both EGFR and KRAS mutation is generally in line with the published literature [20]. The cerebellum has been shown to be the region of predilection from BM depending on vasculature and histology of primary cancer [21,22,23]. It should be noted that our approach was unique in that we focused on BM distribution according to the three common mutations in contrast to prior studies. For example, a study by Takano et al. showed that the BM with EGFR L858R mutation occurred more often in the cerebellum, caudate and temporal lobe than those having EGFR with exon 19 deletion. However, they did not report spatial distribution of BM for ALK or KRAS mutation [8]. A study by Yanagihara et al., reported that 5% to 20% of lung or breast cancer BM occurred in the cerebellum, and BM distribution may follow the patterns of cerebral blood flow [6]. However, their sample size was small, with a total of 28 patients and having only five EGFR+, two KRAS+ and one ALK+ patients in their cohort. They could not evaluate the specific distribution of metastases according to their mutation status [6]. BM in cerebellum has been explained by the retrograde pathways via the Batson venous plexus to the cerebral dural sinuses [24]. However, the venous drainage of lung cancer does not involve the Batson venous plexus but the pulmonary vein into the heart. Therefore, lung cancer BM to cerebellum may not be due to the retrograde venous flow to the brain.

Lung cancer BM reaches the brain through arterial dissemination and may reach the most distal smallest arterioles. Therefore, it is not surprising that BM reaches the distal middle cerebral artery territory involving the precentral and post central gyrus as seen in our study. However, it is unclear why the BM reached one arterial territory rather than other territories and why there was a discrepancy in BM distribution among the groups with different genetic mutations. Distant metastases in general have been explained by Paget’s “seed and soil therapy” [25,26]. This theory indicates that the tumor cells (the “seed”) have a distinct affinity for the microenvironment of a certain organ (the “soil”) which increases the likelihood of metastasis in this region. More recent studies indicate that certain molecular structures such as Neuroserpin and chemokine receptors such as CXR4 and CCR7 may play a role in BM distribution [27,28]. More work needs to be done to understand the mechanism underlying the preferential distribution of BM.

Our EGFR mutation-positive group had both similar and different patterns of BM distribution compared to the study by Takeno et al. [8]. For instance, our EGFR-positive group had BM more frequently located in the cuneus, precuneus, left occipital lobe and cerebellum, which were not reported in their study [8]. Their study reported caudate and temporal lobe as high-frequency regions of BM for the EGFR-positive group [8], but we did not identify these two regions. Yanagihara et al. reported the spatial distribution of BM among NSCLC patients; however, their study did not identify BM based on its mutation status, such as EGFR, ALK or KRAS [6]. The discrepancy in BM distribution patterns between our study and the prior studies may be partly due to our focus on the three mutations and our approaches for frequency analysis against random distribution.

Our finding of preferential BM distribution should have important clinical implications. We showed the preferential distribution of BM for the EGFR group to medial frontal gyrus which may involve memory function and decision making [29,30]. BM for the ALK group had preferential distribution to right precentral and postcentral gyrus, which may be critical for sensory motor function. Better understanding of BM spatial distributions could aid in decision making to employ systemic therapies such as chemotherapy, and local therapies such as radiation and or surgery.

We performed GMD analysis to assess the BM distribution with respect to the brain surface. It should be noted that the GMD computed by SPM was about the gray matter tissue probability at each voxel on the brain image, whose value ranged from 0 to 1. Our results showed that the gray matter probability was 60% at the most likely locations of BM. This finding was consistent with the notion that the likely locations of the BM were the junctions of gray matter and the other two types of brain tissue such as white matter and cerebrospinal fluid. This finding provided complementary information to our volumetric analysis of tumor frequency distribution, which indicated that BM appeared in higher frequency than random distribution at the gray–white matter junction. A prior study by Hwang et al. showed that 64% of BM in their cohort from various primary cancers from lung, breast, skin, kidney, etc. were in the gray–white matter junction. However, they did not perform analysis based on mutation status [31]. Our approach was novel in that the BM was registered into the standard space and our distribution data included details of specific clusters and voxels in the brain through statistical parametric mapping. Therefore, our method was more advantageous with detailed neuroanatomical information while other studies assessed BM distribution through visual estimation.

Characteristics of BM, such as the depth and size of tumor may also influence clinical outcomes. Increasing the depth of tumor may worsen the overall survival and disease-specific survival in patients with BM [32]. A previous study by Takano et al. reported that lesions with the wild-type EGFR mutation were located further away from the brain surface with a tumor depth of 15.0 mm (range: 10.0–20.7 mm) than the L858R EGFR lesions with tumor depth of 13.7 mm (range: 8.6–21.9 mm) [8]. However, the previous studies did not report the median depth of BM in ALK and KRAS mutation subgroups. On the other hand, our study showed the BM of KRAS mutation group (9.1 mm) being located deeper from the brain surface than that of ALK (8.7 mm) and EGFR (7.9 mm) mutation groups. In addition, our study also showed that the KRAS mutation-positive group had the largest tumor size (2.83 ± 6.87 cm^3^) relative to ALK (1.23 ± 3.38 cm^3^) and EGFR (0.51 ± 2.68 cm^3^) mutation groups. Our study implied that the patients with KRAS mutation may have higher risks of an adverse outcome than other two groups because of their larger BM tumors which may involve more brain tissue in eloquent regions and cause more neurological deficits.

Additional characteristics of BM such as peritumoral edema/tumor ratio may affect overall survival. A study by Spanberger et al. reported a strong correlation between the extent of peritumoral edema on brain MRI scans and overall survival, i.e., patients with small peritumoral edema had longer survival [33]. Our previous radiogenomic studies with machine learning models in lung cancer patients with BM showed similar findings, i.e., lower edema/tumor ratio indicating longer survival duration [17,18]. In this study, the ALK mutation group had higher edema/tumor ratio (5.66 ± 21.15) than KRAS (5.08 ± 8.70) and EGFR (2.38 ± 6.13) positive mutation groups, implicating worse survival in the ALK mutation group.

Limitations of this study should be noted. First, this was a retrospective study with inherent limitations of uncontrolled covariables such as primary cancer treatment and brain MRI scan imaging parameters. These variables could potentially contribute to differences in identification and segmentation of BM tumors. Further studies with deep learning-based techniques may improve lesion classification. Second, our sample size was modest, especially for the KRAS mutation-positive group. It was plausible that subtle distribution patterns may not have been detected. Third, we constructed the tumor frequency map based on the notion that all BM tumors could be regressed to the central points as the origins of BM tumors. This notion has been supported by the observation of BM being generally round and showing concentric growth. This method has been used in prior studies of BM [8]. However, we understand that not all BM tumors are round, and they may have irregular shapes, which may affect the identification of the central points for BM.

## 5. Conclusions

In summary, our study showed the preferential distribution of lung cancer BM to the left cerebellum, bilateral medial frontal gyrus, right precentral gyrus and right postcentral gyrus, according to mutation status. Our study should motivate further research to understand the mechanism of lung cancer spreading to the brain. It should also be potentially useful in the personalized treatment planning of BM as to preserve brain structure and function for patients with cancer and metastatic disease.

## Figures and Tables

**Table 1:** Clinical data and tumor information of the participants.

	EGFR (+)	ALK (+)	KRAS (+)	*p*-Value
	*n* = 75	*n* = 21	*n* = 15	
**Age (years)**				
Mean ± SD	57.43 ± 12.09	53.81 ± 14.79	63.67 ± 6.40	0.09
**Gender**				
Male	24 (32%)	8 (38.10%)	5 (33.33%)	0.83
Female	51 (68%)	13 (61.90%)	10 (66.67%)	
**Race**				
Caucasian	34 (45.33%)	13(61.90%)	11 (73.33%)	0.016
Asian	35 (46.67%)	7 (33.33%)	1 (6.67%)	
Other ^1^	6 (8%)	1 (0.04%)	3 (20%)	
**History of Smoking**				
Yes	20 (26.67%)	5 (23.80%)	12 (80%)	<0.001
No	55 (73.33%)	16 (76.19%)	3 (20%)	
**Histology**				
Adenocarcinoma	71 (94.67%)	21 (100%)	13 (86.67%)	0.5
Other ^2^	4 (5.33%)	0 (0%)	2 (13.33%)	
**Number of tumors**	15.79 ± 35.24	9.07 ± 14.01	6.18 ± 7.41	0.34
**Tumor Size (cm^3^)**	0.51 ± 2.68	1.23 ± 3.38	2.83 ± 6.87	<0.0001
**Median Tumor Depth (mm)**	7.9	8.7	9.1	0.044
**Edema/Tumor Volume Ratio**	2.38 ± 6.13	5.66 ± 21.15	5.08 ± 8.70	<0.0001

Abbreviations: EGFR, epidermal growth factor receptor; ALK, anaplastic lymphoma kinase; KRAS, v-Ki-ras2 Kirsten rat sarcoma viral oncogene. Other ^1^: American Indian or Alaska Native, African American, Native Hawaiian, or Pacific Islander; Other ^2^: Squamous cell lung carcinoma, adenosquamous cell lung cancer, lung carcinosarcoma, non-small cell lung carcinoma (NOS).

**Table 2:** Brain regions where brain metastases occurred at a higher frequency than uniform distribution for each of the three mutation positive groups.

Group	Center (MNI)	Cluster Size	*p* Value	Brain Region
EGFR	-28.5_-63.0_-33.0	6060	0.0002	L Cerebellum, Anterior Lobe, Culmen
	-30.0_-61.5_-40.5	6060	0.0002	L Cerebellum, Posterior Lobe, Cerebellar Tonsil
	-18.0_-66.0_-28.5	6060	0.0002	L Cerebellum, Posterior Lobe, Declive
	-16.5_-61.5_-31.5	6060	0.0002	L Cerebellum, Anterior Lobe, Dentate
	-13.5_-73.5_31.5	5433	0.0006	L Occipital Lobe, Cuneus
	-12.0_-76.5_21.0	5433	0.0006	L Occipital Lobe, Cuneus (BA 18)
	-9.0_-75.0_21.0	5433	0.0006	L Parietal Lobe, Precuneus (BA 31)
	-15.0_-69.0_24.0	5433	0.0006	L Parietal Lobe, Precuneus
	36.0_-16.5_49.5	23,128	0.0001	R Frontal Lobe, Precentral Gyrus
	28.5_-19.5_63.0	23,128	0.0001	R Frontal Lobe, Precentral Gyrus (BA 6)
ALK	28.5_-79.5_13.5	1576	0.022	R Occipital Lobe, Middle Occipital Gyrus
	25.5_-66.0_10.5	1576	0.022	R Limbic Lobe, Posterior Cingulate
	21.0_-78.0_21.0	1576	0.022	R Parietal Lobe, Precuneus (BA 18)
	37.5_-16.5_51.0	8814	0.0001	R Frontal Lobe, Precentral Gyrus (BA 4)
	40.5_-19.5_54.0	8814	0.0001	R Frontal Lobe, Precentral Gyrus
	37.5_-31.5_43.5	8814	0.0001	R Parietal Lobe, Inferior Parietal Lobule (BA 40)
	40.5_-33.0_45.0	8814	0.0001	R Parietal Lobe, Postcentral Gyrus
KRAS	-24.0_-72.0_-30.0	1496	0.0278	L Cerebellum, Posterior Lobe

Abbreviations: EGFR, epidermal growth factor receptor; ALK, anaplastic lymphoma kinase; KRAS, v-Ki-ras2 Kirsten rat sarcoma viral oncogene. L, left; R, right; BA, Brodmann area.

## Data Availability

The datasets generated for this study are available on request to the corresponding author.

## Supplementary Materials

The following supporting information can be downloaded at: https://www.mdpi.com/article/10.3390/brainsci13071057/s1, Figure S1: Depth of the tumor center from the brain surface for EGFR, ALK, and KRAS mutation groups; Figure S2: Distribution of the gray matter density (GMD) of brain metastasis for EGFR, ALK, and KRAS mutation groups indicating most frequent location of brain metastasis at the gray - white matter junction (GMD for all three groups peaked at 0.6); Table S1: The voxel-level and cluster-level thresholds to identify the brain regions where the brain metastasis frequency being higher than random distribution.
