# Supplementary material for: Differential Distribution of Brain Metastases from Non-Small Cell Lung Cancer Based on Mutation Status
Source: Brain Sci. 2023 Jul 11;13(7):1057. doi: 10.3390/brainsci13071057 (PMC10377121; doi:10.3390/brainsci13071057)

Supplementary files:

Table S1. The voxel-level and cluster-level thresholds to identify the brain regions where the brain metastasis frequency being higher than random distribution. The brain regions were identified through a two-step process: 1) applying the single voxel level threshold to construct clusters where the brain metastasis frequency at each voxel was higher than uniform distribution with significance level p=0.001 without adjusting for multiple comparison. 2) applying the cluster level threshold to identify the clusters where the brain metastasis frequencies were higher than uniform distribution with cluster-level p value less than 0.05 after non-parametric multiple comparison adjustment. Abbreviations: EGFR, Epidermal growth factor receptor; ALK, Anaplastic lymphoma kinase; KRAS, v-Ki-ras2 Kirsten rat sarcoma viral oncogene.

Group	Cutoff (voxel)	Cutoff (cluster)
**EGFR**	0.0256	1769
**ALK**	0.0497	1049
**KRAS**	0.0619	1061

Figure S1. Depth of the tumor center from the brain surface for EGFR, ALK, and KRAS mutation groups. EGFR, epidermal growth factor receptor; ALK, anaplastic lymphoma kinase; KRAS, v-Ki-ras2 Kirsten rat sarcoma viral oncogene.

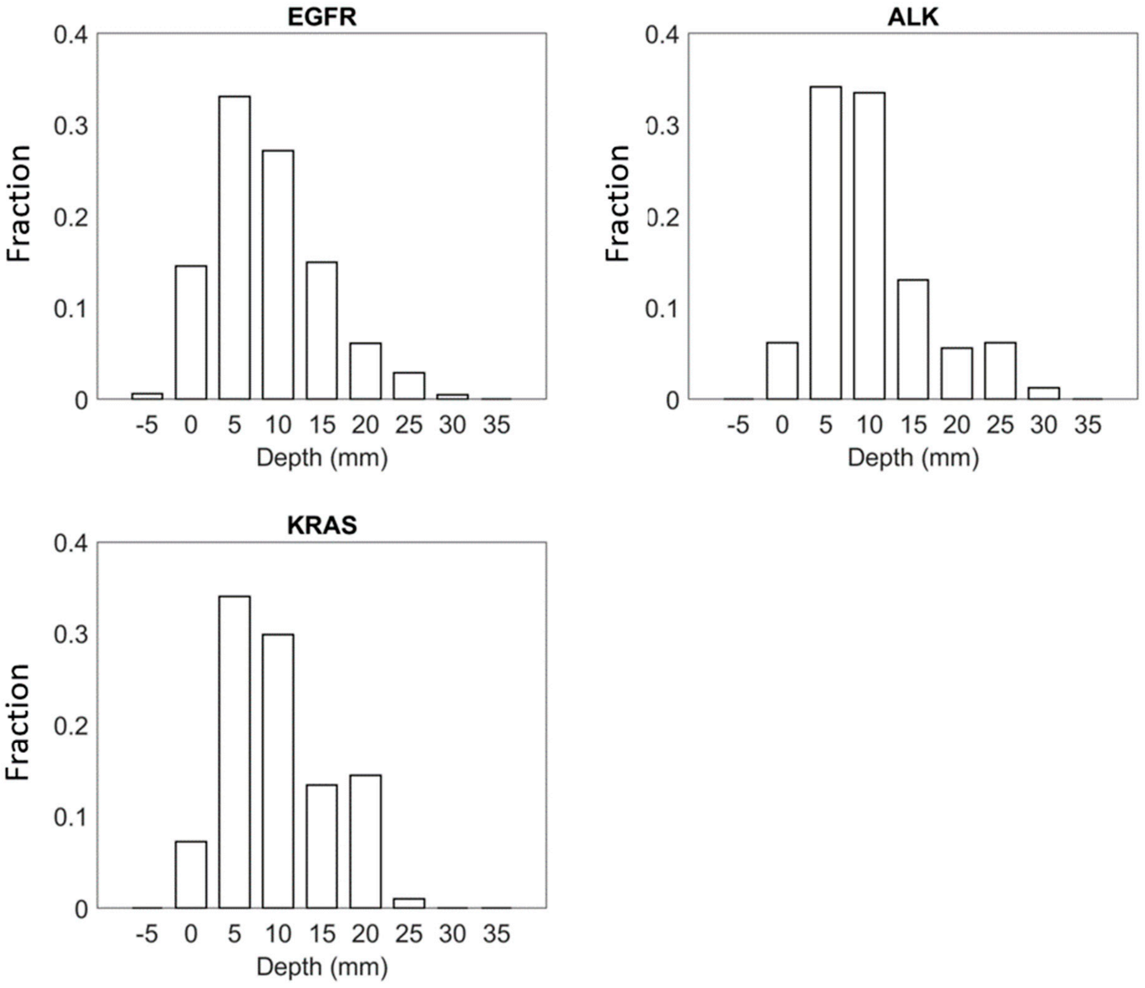

Figure S2. Distribution of the gray matter density (GMD) of brain metastasis for EGFR, ALK, and KRAS mutation groups indicating most frequent location of brain metastasis at the gray - white matter junction (GMD for all three groups peaked at 0.6). EGFR, epidermal growth factor receptor; ALK, anaplastic lymphoma kinase; KRAS, v-Ki-ras2 Kirsten rat sarcoma viral oncogene.

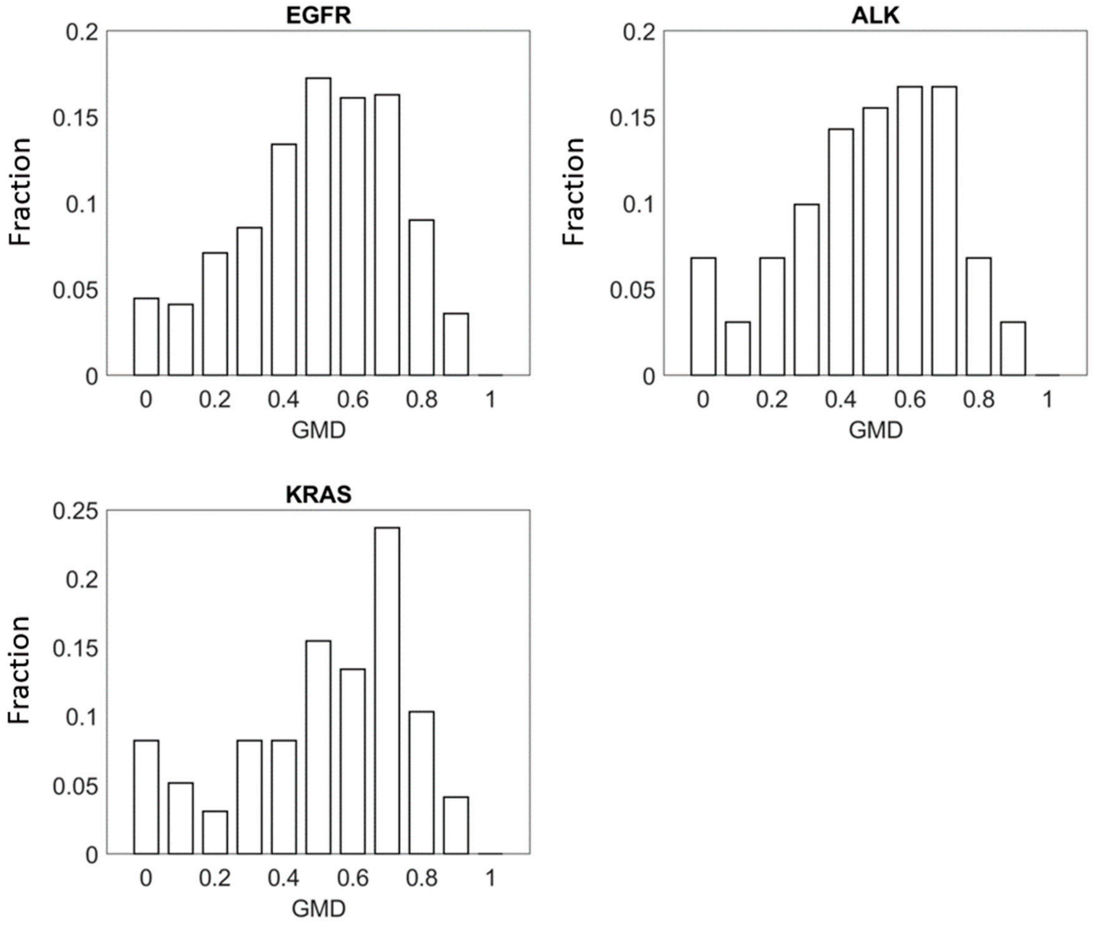

Supplement: Supplementary file 1 [file brainsci-13-01057-s001.zip › brainsci-2495678-SI.pdf]
